# Gradient Index Microlens Implanted in Prefrontal Cortex of Mouse Does Not Affect Behavioral Test Performance over Time

**DOI:** 10.1371/journal.pone.0146533

**Published:** 2016-01-22

**Authors:** Seon A. Lee, Kevin S. Holly, Vladislav Voziyanov, Stephanie L. Villalba, Rudi Tong, Holly E. Grigsby, Edward Glasscock, Francis G. Szele, Ioannis Vlachos, Teresa A. Murray

**Affiliations:** 1 Center for Biomedical Research and Rehabilitation Sciences, Louisiana Tech University, Ruston, Louisiana, United States of America; 2 Department of Cellular Biology and Anatomy, Louisiana State University Health Sciences Center, Shreveport, LA, United States of America; 3 Department of Physiology, Anatomy and Genetics, University of Oxford, Oxford, United Kingdom; Radboud University Medical Centre, NETHERLANDS

## Abstract

Implanted gradient index lenses have extended the reach of standard multiphoton microscopy from the upper layers of the mouse cortex to the lower cortical layers and even subcortical regions. These lenses have the clarity to visualize dynamic activities, such as calcium transients, with subcellular and millisecond resolution and the stability to facilitate repeated imaging over weeks and months. In addition, behavioral tests can be used to correlate performance with observed changes in network function and structure that occur over time. Yet, this raises the questions, does an implanted microlens have an effect on behavioral tests, and if so, what is the extent of the effect? To answer these questions, we compared the performance of three groups of mice in three common behavioral tests. A gradient index lens was implanted in the prefrontal cortex of experimental mice. We compared their performance with mice that had either a cranial window or a sham surgery. Three presurgical and five postsurgical sets of behavioral tests were performed over seven weeks. Behavioral tests included rotarod, foot fault, and Morris water maze. No significant differences were found between the three groups, suggesting that microlens implantation did not affect performance. The results for the current study clear the way for combining behavioral studies with gradient index lens imaging in the prefrontal cortex, and potentially other regions of the mouse brain, to study structural, functional, and behavioral relationships in the brain.

## Introduction

Mouse behavioral experiments with complementary approaches such as genetic knockouts of receptors, and pharmacological or optogenetic manipulation of circuits, have played a critically important role in investigating conditions such as traumatic brain injury [1, 2], addiction [3, 4], mood disorders [5–7], and neurodegenerative diseases [8–10]. More recently, *in vivo* imaging has allowed us to peer into the intact brain of a mouse and observe dynamic cellular activities, such as microglial processes moving in the neuropil in the cerebral cortex [11], and subsecond network activity in a behaving mouse through calcium-sensitive dyes and genetically encoded calcium indicators [12, 13]. Two important tools for advancing *in vivo* imaging in rodent models are the multiphoton laser scanning fluorescence microscope, which can resolve cells up to several 100-μm below the pial surface [11, 14, 15], and cell-type-specific, promoter-directed expression of fluorescent proteins [11, 16, 17]. These technologies have paved the way for *in vivo* imaging of subpopulations of neurons, astrocytes, and microglia [11, 16–18]. However, most cells reside too deep within the brain to observe with multiphoton microscopy [18]. In contrast, magnetic resonance imaging and computed tomography can visualize the entire brain. Yet, they do not have the cellular and temporal resolution to capture dynamic activity of individual cells [19, 20]. Other techniques, such as chronically implanted multielectrode arrays [21] and optogenetics [22] enable recording and control, respectively, of large numbers of neurons in deep regions of the brain [23, 24]. However, the relationship of active cells within these networks are not resolved and electrochemically inactive cells are not detected [13]. As such, most research into spatio-temporal activity patterns of cells in local deep-brain networks relies on brain slices [16, 25] and cultured cells [26–28]. However, an inherent limitation of such "reduced preparations" is that it is difficult to be certain how accurately they represent *in vivo* activity. To circumvent this problem, an emerging technique employing implanted gradient index (GRIN) lenses has extended the reach of optical microscopy to deep cortical [14, 29] and subcortical regions [18, 30] of the intact mouse brain, overcoming the spatiotemporal limitations of these other modes of investigation [13].

Early iterations of the GRIN lens were composed of two or more lenses, which were relatively wide and long (≥ 1 cm) compared to current designs. Due to their large size, they were used for acute imaging of neurons labeled with fluorescent protein and for determining microvascular blood flow rates in a very small region of a few tens of microns in diameter [14, 29]. A more recent design uses a thinner, shorter lens that is flush with the skull and has a four-fold increase in the field of view, to about 200 μm [18]. This is a wide enough field of view to observe local networks [31]. Additionally, the low profile lens is not predicted to interfere with feeding, grooming, and other activities, such as behavioral tests.

Pairing deep brain imaging with behavioral tests and other modes of noninvasive imaging would be a powerful combination of investigative techniques for advancing neuroscience research. By analyzing GRIN lens images over time, behavioral performance could be correlated with cellular and microvascular changes in a target brain region and with more global changes using noninvasive imaging of the whole brain. However, the effect of lens implantation on behavioral tests has not been studied in controlled experiments. Given the small diameter of the newer lens, it was hypothesized that its implantation would not have a significant effect on commonly-used behavioral tests of integrated motor ability, locomotor ability, and spatial learning and memory [32]. The present study shows that chronic GRIN lens implantation into the prefrontal cortex of mice does not cause deficits in common behavioral tests, including rotarod, foot fault, and Morris water maze tests.

## Materials and Methods

### Ethics statement

A protocol describing all housing and behavioral procedures was approved by the Louisiana Tech University Institutional Animal Care and Use Committee before experiments were conducted. Furthermore, all procedures were in accordance with the [United States] National Institute of Health Guide for the Care and Use of Laboratory Animals. All mice used in this project were housed in the OLAW and USDA approved housing facilities of the Center for Biomedical Engineering and Rehabilitation Sciences, Louisiana Tech University. All surgeries were performed under isoflurane anesthesia, with an initial dose of ketamine and xylazine, and every effort was made to minimize pain and suffering.

### Care and use of mice

Food and water was provided *ad libitum* and mice were housed with a twelve-hour light/dark cycle. Transgenic mice expressing green fluorescent protein (GFP) under the human glial fibrillary acidic protein (GFAP) promoter (FVB/N-Tg(GFAPGFP)14Mes/J strain, Jackson Labs, jaxmice.jax.org/strain/003257.html) were used to visualize astrocytes (n = 12). Wild type (WT) littermates (n = 3) were also used. Adult mice of mixed gender (male n = 8; female n = 7) were used for experiments. Mice were genotyped by RealTime Laboratories, Inc. (Carrollton, Texas, USA). The effects of gender and transgenic status were assessed and no significant differences were found (Table 1). Mice were housed in groups of 2–4 per cage, and all cages were located in the same housing room.

**Table 1:** Significance of factors in presurgical tests.

Test \ Factor	Time	Gender	GFAP-GFP +/-
Rotarod	0.002	0.229	0.112
Foot Fault	0.091	0.368	0.978
MWM T1	0.939	0.876	0.896
MWM T2	0.517	0.192	0.589

Table contains p-values for repeated measures factorial ANOVA performed for each test. (MWM is the Morris water maze, T1 is the time to reach the platform, and T2 is the swim speed).

### Assignment to treatment groups and surgical procedures

**Fig 1:**
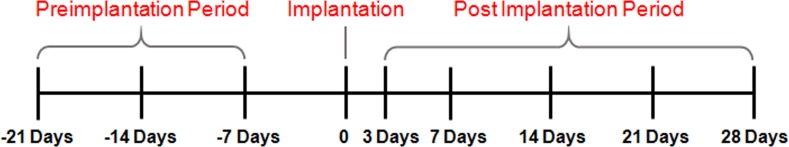
Experimental timeline. Three weekly sets of behavioral experiments were performed prior to surgery to establish baseline performance for each mouse. After this, mice were randomly assigned to one of three treatment groups: GRIN Lens implant, cranial window (No Lens), or sham surgery (Anesthesia Only). No behavioral experiments were performed on the day of surgery. Post implantation experiments were run 3 days after surgery to implant a GRIN lens, install a cranial window, or conduct a sham surgery to determine the acute effects of lens implantation, craniotomy surgery, and anesthesia, respectively. Tests were conducted weekly at 7, 14, 21, and 28 days after surgery to determine the time course of effects due to chronic implantation, if any. Each mouse was tested at each of the 8 time points for all three behavioral tests (n = 5 mice for each treatment group).

After the first three weeks of behavioral tests, mice were randomly assigned to one of three treatment groups by assigning a number to each animal and then blindly drawing twice-folded pieces of numbered paper from an opaque container. Random assignment to treatment groups were as follows: (1) “GRIN Lens,” a lens implant with cranial window (2 GFP+ males, 1 WT male, 2 GFP+ females), (2) “No Lens,” a cranial window only (1 GFP+ male, 1 WT male, 1 GFP+ female), and (3) “Anesthesia Only,” a sham surgery with no scalp incision or craniotomy, but using the same method of anesthesia, head restraint, and duration as the other two surgeries (1 WT male, 4 GFP+ females). No mice were excluded at any time; each mouse was used for all three behavioral tests at all 8 time points (Fig 1).

Prior to GRIN lens implantation, a singlet GRIN lens 350 μm in diameter (Go!Foton Corp.) was attached to a cranial window as previously described [18]. Cranial windows were composed of a 3-mm, number 1.5 glass cover slip (Electron Microscopy Sciences) and a 5-mm wide x 1-mm thick stainless steel washer was affixed to the top of the cover glass. The washer facilitated rapid identification of the implanted lens for imaging. (It should be noted that the steel washer can be replaced with a nylon washer if steel would interfere with the use of other imaging modalities.) The cover slip and washer were attached to a Narishige EDMS13-392 12 mm x 12 mm x 1 mm aluminum head plate. The head plate was machined in-house with a cut-out for mounting the lens, cover slip and washer. (Narishige will custom cut their head plates upon request.) Since this test was conducted, all-polymer head plates for compatibility with non-invasive imaging were designed and tested. These performed as well as the aluminum plates.

Aseptic surgery to implant the lens was performed as previously described [18] with three exceptions, as follows: (1) Mice were initially anesthetized by isoflurane (5%) then given a ketamine/xylazine cocktail intraperitoneally (10 mg/kg ketamine and 1 mg/kg xylazine) to facilitate positioning into the stereotaxic frame. They were maintained throughout surgery on 1.5%–1.8% isoflurane. (2) The lens was implanted in the prefrontal cortex, 2.5 mm rostral to bregma and 1.25 mm lateral to the midline in the left hemisphere to a depth of 0.9 mm. This placed the rostral -most side of the lens through the interface of the motor cortex and the frontal association cortex with the end of the lens in the frontal association cortex [33]. (3) Cranial windows were secured to the skull by RelyX dental adhesive (3M Corp.) instead of dental acrylic. Surgeries to install cranial windows were the same as those performed to implant GRIN lenses. Sham surgeries used the same methods of anesthesia for the same duration as GRIN lens implantations and cranial window installations but no surgical procedures were done (i.e., no scalp incision and no craniotomy).

To ensure that animals felt no pain during surgical procedures, toe pinches were performed every 15 minutes to ensure that the animals were fully anesthetized. To minimize post-surgical pain, an analgesic was dissolved in the animal’s drinking water for 60–72 hours following surgery (Ibuprophen, children’s suspension, 30 mg/kg/day). After the last set of behavioral experiments at 28 days after surgery, mice were euthanized using a CO_2_ chamber. No animals used in this study died prematurely. Animals were monitored seven days per week and all were reported in good health.

### Behavioral test schedule

Mice were tested weekly for three weeks prior to surgery (Day -21, -14, and -7) to acquire baseline measurements and to account for the effects of task learning over time for all three behavioral tests. Mice were tested in the same order each day between 11 am–3pm, with one exception. For Day -7, the Rotarod and Foot Fault tests were conducted between 6–8 pm. After three weeks, mice were randomly assigned to one of the three treatment groups (Fig 1). Behavioral tests were performed three days after surgery (Day 3) to determine the acute effects of the GRIN lens implant, the effects of the craniotomy surgery (No Lens), and the effect of anesthesia, respectively. The same mice performed behavioral tests weekly for four weeks after surgery at 7, 14, 21, and 28 days after surgery to assess the effects of chronic implantation. For all tests, mice were brought into the testing room and the filter cover of the cage was removed for 15 minutes to acclimate the animal to the room [34]. A 15-minute break was given between tests. The behavioral tests were performed in quiet rooms in the laboratory.

### Rotarod test

The standard operating procedure, ESLIM-010-001 Revision Number 2, from the European Mouse Phenotyping Resource of Standardized Screens, or EMPReSS, was used for conducting the rotarod test. Sensors were located on the landing platform. When the mouse fell into the landing area, a sensor was triggered which stopped a digital timer, thus displaying the mouse’s latency to fall [35]. Briefly, three trials were performed, with a 15-minute interval in between trials, for each testing period. The mean latency to fall was calculated for each mouse for each testing period.

### Foot fault test

A foot fault test, or grid walk test, was used to assess motor coordination skills. A 25.4 cm x 40.6 cm wire rack consisting of 1 mm diameter metal wire in an 8 mm x 8 mm grid was used as the walk platform. Mice were placed on the elevated walk platform and allowed to roam freely for 3 minutes. The web cam of a tablet personal computer (ASUS TF300t, ASUSTeK Computer Inc.) was placed under the platform to record 50 steps on the grid, or for 3 minutes, whichever was less. The video was analyzed offline to quantify the number of each limb’s “foot slips” through the grid. A “foot slip” was counted when the limb missed the footing or failed to bear the weight of the mouse [36]. Analysis of the videos was performed by persons who were blinded to the treatment condition.

### Morris water maze test

The Morris water maze (MWM) test was used to assess cognitive ability and swim speed. It was performed as described previously [37]. Briefly, a platform was hidden under the water in a fixed quadrant. The starting position of the mice was alternated until all four quadrants were used. Mice were given a 5-day training period to learn the task. If a mouse failed to find the platform within the allotted time for two trials out of the four on given day, an additional day of training was administered until the mouse successfully completed at least three of the four trials before being used for experiments. For acquiring video of each trial for offline analysis, a metal frame was constructed above the tub to mount an ASUS tablet PC. Experimental outcomes were the time to find the platform and the average swim speed (distance traveled divided by time, in cm/s). Analysis of the videos was performed by persons who were blinded to the treatment condition.

### *In vivo* imaging

Mice were anesthetized using 5% isoflurane and then maintained on 1.5%–1.8% isoflurane for imaging. After anesthesia was induced, the head plate was inserted into a Narishige Model MAG-1 head plate holder (Narishige International USA, Inc.). Scanned images of GFP-labeled astrocytes in layer 5 of the frontal association cortex were acquired using an upright, Vivo 2-Photon microscope with GaAsP detectors (Intelligent Imaging Innovations, Inc.), a green fluorescent protein filter set (Semrock, Inc.), a Chameleon multiphoton laser (Coherent) tuned to 900 nm, and an ELWD 40X, 0.6 numerical microscope objective (Nikon) with a correction collar. Background noise was subtracted in Slidebook 6 (Intelligent Imaging Innovations, Inc.) and exported as 16-bit TIFF files. Image J software was used to make a z-projection of two adjacent image planes that were 1 μm apart [38].

### Histology

#### Perfusion and fixation of brain tissue

Mice were deeply anesthetized with ketamine/xylazine (100 mg/kg, 10 mg/kg) via an intraperitoneal injection, and then perfused transcardially with phosphate buffered saline (PBS) and then 20 ml of 4% paraformaldehyde solution, 7.2 pH, at 4°C (PFA). The heads were decapitated and post-fixed in PFA for 4 hours prior to removing the lens from the brain and the brain from the skull. Lenses were examined microscopically to confirm that no tissue was attached to the end of the lens. If tissue was attached to the end of the lens, that brain was not processed for histology. After dissection, brains (n = 3) were immersed in 30% sucrose in PBS for cryopreservation prior to slicing, as described previously [39].

#### Immunohistochemistry for GFAP and imaging

Mouse brains, as processed above, were embedded in OCT (optimal cutting temperature) medium and frozen at -80°C. Brains were sectioned at 30 μm using a cryostat held at -20°C and the sections collected on charged slides for immunofluorescence. Tissue sections were allowed to dry for 1 h and then were washed with PBS. Background staining was blocked with 10% bovine serum albumin (BSA) in 0.3% Triton in PBS for 1 h at room temperature (RT; ∼22°C). Sections were incubated with the primary antibody, mouse anti-glial fibrillary acidic protein (GFAP) (clone N206A/8; 1:500 dilution in blocking solution; NIH Neuromab Facility, UC Davis, USA) overnight at RT. Following incubation with primary antibody, sections were washed in blocking solution and incubated for 1 h at RT with the secondary antibody, Alexa Fluor 488 goat anti-mouse IgG1 (1:1000 dilution in blocking solution; Life Technologies, Waltham, MA, USA). Following incubation with the secondary antibody, sections were washed first with blocking solution and then with PBS, and allowed to dry at RT in the dark. Coverslips were mounted using ProLong® Diamond Antifade mounting solution with DAPI (Life Technologies, Waltham, MA, USA) and allowed to cure overnight at RT in the dark [39].

Stained tissue was imaged using an Olympus IX51 inverted microscope with an X-Cite Series 120 illumination system and a green fluorescence emission filter. The tissue slice with the widest lens track (middle of lens) was chosen for further analysis. The lens track width varied because the lens is cylindrical. When the tissue is cut in coronal sections, the widest hole includes the location of the center of the lens. The lens track is rectangular in shape because the GRIN lens has a flat bottom. A region in the ventrolateral cortex of the contralateral hemisphere (contralateral control) was also imaged in each slice. Images were captured as 1360 x 1024 pixel jpg or tif files using a DP71 camera with the DP Manager software program (Olympus). An integration time of 0.167 second was used for each image. Images were stored on the microscope system hard drive and also on external hard drives.

#### Immunohistochemistry for immune cells and imaging

Immunohistochemistry was carried out as in [40–42]. Briefly, for immunohistological processing, 30 μm-thick free-floating coronal sections for were made on a sliding microtome (Leica SM2000R) and stored in cryoprotectant (0.25 M phosphate buffer, 30%w/v sucrose, 5.38 M ethylene glycol) at -20°C. For immunohistochemistry, sections were washed in PBS three times for 10 min, then for 15 min in 50 mM glycine dissolved in PBS and then washed again in PBS three times for 10 min. After this, sections were incubated in PBS+ (10% donkey serum and 0.1% Triton X-100 in PBS) for 1 h. Sections were incubated overnight at 4°C in primary antibody diluted in PBS+ (anti-Iba1, 1:200, goat polyclonal, Abcam ab5076). Sections were washed three times in PBS, incubated with AlexaFluor-conjugated secondary antibody (Alexa Fluor 568, Life Technologies) diluted 1:500 in PBS+ for 1 h, washed again three times in PBS and counterstained with DAPI (MP Biomedicals, 10 μg/ml). Afterward, slices were washed in 0.1 M phosphate buffer and mounted onto a coverslip (mounting medium: FluorSave^TM^, Millipore 345789).

Fluorescence images were acquired using a Leica DMIRB inverted microscope, with a 20x/0.50 NA air objective. Images were captured as 16-bit TIFF files using Volocity^TM^ Software (Perkin-Elmer).

### Image Analysis

Image analysis for determining the thickness of the glial scar was performed using Image J software [38] on original images without adjustments for brightness or contrast. Using the rectangular Region of Interest tool, a long thin box was drawn under the lens track and placed at one end of the track. An intensity profile was measured using the plot profile tool. The box was then moved to the adjacent, non-overlapping area and another intensity profile was acquired. This was repeated until intensity profiles were acquired for the entire region under the lens track (3 mice, total of 40 areas). A sharp increase in intensity occurred in each area near the lens implant denoting an increase in the level of GFAP. The baseline intensity was determined for each plot. This was set below the peaks from stained GFAP+ cells. It also did not include the brighter, more densely stained area next to the lens track in the tissue. A straight line was drawn across the plot at the baseline intensity. A second line was drawn near the end of the lens track where the baseline fluorescence intensity increased rapidly. Due to the rapidly increasing intensity, this line was angled sharply upward. The intersection of this line with the previously scribed baseline level was used to define the “edge” of the glial scar. The distance between this intersection and the end of the lens track in the tissue was used to measure the thickness of the glial scar. Additionally, the numbers of GFAP+ cells were also compared (see S1 Dataset). A two-tailed, unpaired Student’s t-test was used to compare the number of GFAP+ cells in the non-scar areas under the lens with a control area in the ventrolateral cortex in the contralateral hemisphere. The level of significance was set to α = 0.05.

Fluorescence images of coronal brain slices labeled with anti-Iba1 and counterstained with DAPI were acquired using a Leica DMIRB inverted microscope, with a 20x/0.50 NA air objective. Images were captured as 16-bit TIFF files using Volocity^TM^ Software (Perkin-Elmer). Image J was used to define and measure the area (μm^2^) of two regions of interest (ROI) under the lens. One ROI encompassed the area that is too close to the lens to image (non-imaging area*) and the other ROI comprised the area that is imaged using the GRIN lens (imaging area). (*The GRIN lenses used in this study had a working distance of 125 μm, which means that objects less than 125 μm from the end of the lens do not appear in images.) Iba1+ cells with de-ramified (bushy) and amoeboid morphology, typical of activated microglia [1, 11], were counted in each area. A two-tailed, unpaired Student’s t-test was used to compare the number of cells with activated morphology in the non-imaging area with the number in the imaging area. Additionally, two-tailed, unpaired Student’s t-tests were used for comparison of the number of Iba1+ cells with activated morphology in the (1) non-imaging area with a control area in the ventrolateral cortex in the contralateral hemisphere, and the (2) imaging area with the control area. The level of significance was set to α = 0.05. Additionally, the numbers of cells with activated morphology in the non-imaging area that were within 46 μm of the lens were counted. This distance matched the mean thickness of the glial scar.

### Analysis of behavioral tests

The mean value was calculated from individual mouse performance measures for each behavior time point for each test. Repeated measures factorial ANOVA procedures were conducted to compare the effects of gender, genotype (GFP+ vs. GFP -), time and surgical treatment (GRIN lens implant vs. cranial window vs. sham surgery) on the animal’s performance metrics (latency to fall for the rotarod test; foot slip count for the foot fault test; time to find the platform and swim speed for the Morris water maze test). The level of significance was set to α = 0.05. Analyses of the videos of behavioral tests were performed by persons who were blinded to treatment methods.

For the given experimental design, sample size (n = 5 per treatment group), statistical significance level (α = 0.05), and a difference in performance between the treatment groups of up to 15% (partial η^2^ = 0.33), the achieved statistical power was above 80% for all behavioral tests conducted. Given the value of this longitudinal approach for basic science and preclinical research, a slight impairment in behavioral performance would not lessen the beneficial information obtained. If an effect of this size or smaller should occur, it can be controlled or compensated for without diminishing the value of the experiments.

## Results

### Effects of time, sex, and genetic status

Presurgical behavioral performance, which included the first three weekly testing sessions, were used to compare the effects of time, gender, and genetic status (GFAP-GFP+ or GFAP-GFP-). Performance measures were latency to fall in the rotarod test, the number of faults in the foot fault test, and both the swim speed and the length of time to find the platform in the Morris water maze test. Performance for each animal was normalized to its first trial performance for each type of test to minimize the effect differences in ability between animals. The effects of time, gender and genetics (factors) for each behavioral test were evaluated using repeated measures factorial ANOVA. The resulting p values are listed in Table 1. Time was a significant factor in the rotarod test. The mean time to fall increased over the three weekly tests, indicating a learning effect. There was a moderate learning effect in the foot fault test that was not significant at the p < 0.05 level. For the Morris water maze test, no significant differences were found for the effect of time in the length of time taken to reach the platform (MWM, T1, p = 0.939) or in swim speed (MWM T2, p = 0.517). Additionally, no differences were found in performance based on gender or genetic status for any of the behavioral tests.

### Rotarod performance

**Fig 2:**
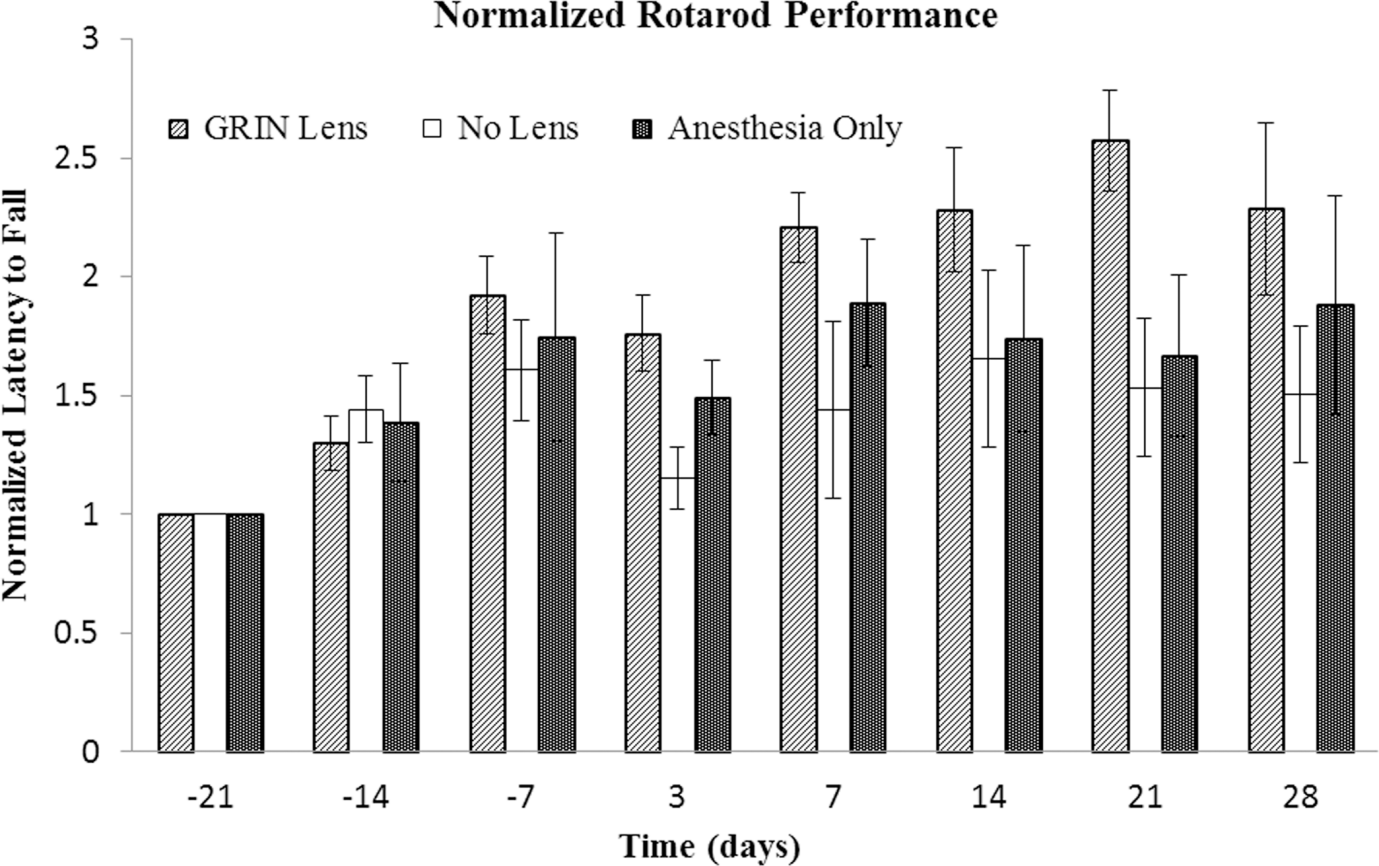
Comparison of performance on rotarod test. Mean latency to fall times for each mouse were normalized to the first week of testing (Day -21). Bars indicate the standard error of the mean. Three tests were performed prior to surgery at -21, -14, and -7 days prior to surgery and then 3, 7, 14, 21 and 28 days after surgery. No significant differences were observed between treatment groups (p = 0.26), which include GRIN lens implant (GRIN Lens), cranial window (No Lens), and sham surgery (Anesthesia Only) groups (n = 5 mice per treatment group). Only the effect of time was significant (p = 0.0002). The upward trend in latency times suggests a learning effect.

The average times for the latency to fall from the rotarod increased from the first testing day (Day -21 test, three weeks prior to surgery) through the next two weeks (Day -14 and Day -7 tests). After this, latency times varied around the values observed during the third testing period, Day -7, regardless of the surgical treatment. Some variability was evident between the individual performance times of mice from the first day of testing and throughout the course of the experiments. To control for this innate difference in ability between mice, the latency times for each mouse were normalized to its mean latency in its first trial (Day -21 test). No differences in latency times existed between mice with GRIN lens implants, cranial windows or sham surgery (p = 0.26). A slight decrease in mean latency time was observed for all three groups in the test that occurred three days after surgery compared to the test 7 days prior to surgery (Fig 2). There was no significant difference between any of the three treatment groups at this time point (p = 0.25), suggesting that this temporary decrease was due to residual effects of anesthesia [43] rather than the type of surgical procedure.

### Foot fault test

**Fig 3:**
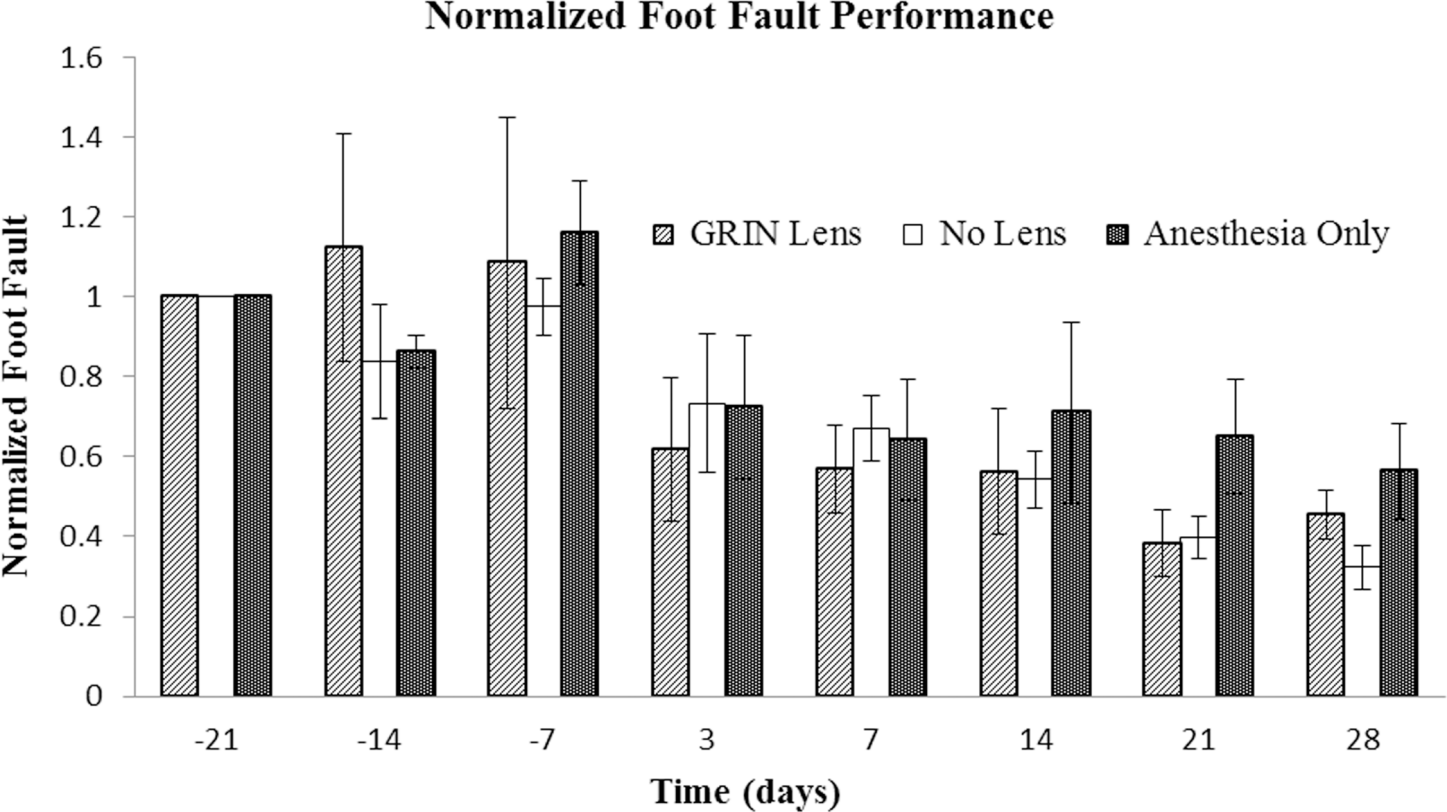
Comparison of performance in foot fault test. The performance of each mouse was normalized to its Day -21 performance. Bars are the standard error of the mean for each treatment group (n = 5 mice per treatment group). Tests were conducted -21, -14, and -7 days prior to surgery and then 3, 7, 14, 21, and 28 days after surgery. Time was the only factor with a significant difference (p = 1 x 10^−5^). The downward trend in the number of faults suggests a learning effect. No significant difference was found between treatment groups (p = 0.78).

Step errors were counted for each mouse for each weekly trial. No significant differences were found between the treatment groups. As with the other behavioral tests, some variability between the individual performance measures in this test was evident. To mitigate the impact of individual variability in abilities on detecting differences between groups, the number of foot faults for each mouse for each trial was normalized to its number of foot faults on the first day of testing (Day -21). No differences in the mean number of foot faults existed between mice with GRIN lens implants, cranial windows or sham surgery (Fig 3, p = 0.60). Unlike the rotarod test, there was no temporary decline in performance after surgery. Time was a significant factor (p = 0.0002). A large decrease in the number of faults is apparent between the test 7 days prior to surgery and 3 days after surgery. This downward trend in the number of faults over time suggests a learning effect as the mice become more proficient at this task.

### Morris water maze

**Fig 4:**
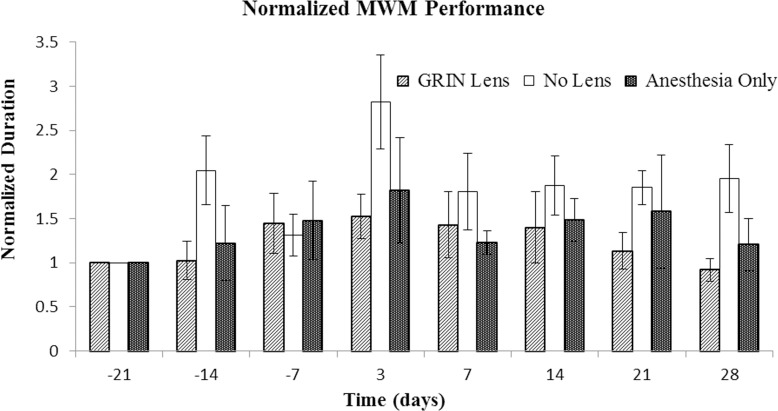
Comparison of time to find the platform for Morris water maze test. The performance of each mouse was normalized to its Day -21 performance. Bars denote the standard error of the mean for each treatment group (n = 5 mice per treatment group). Tests were conducted -21, -14, and -7 days prior to surgery and then 3, 7, 14, 21, and 28 days after surgery. There was no significant difference between treatment groups (p = 0.23).

**Fig 5:**
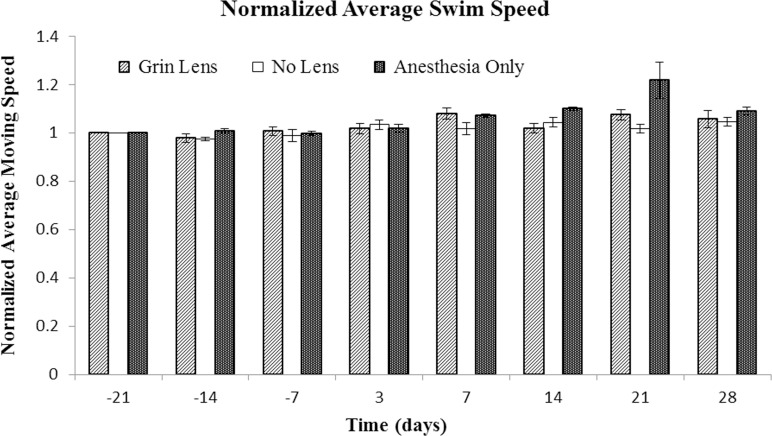
Comparison of swim speed in the Morris water maze test. The performance of each mouse was normalized to its Day -21 performance. Bars are the standard error of the mean for each treatment group (n = 5 mice per treatment group). Tests were conducted -21, -14, and -7 prior to surgery and then 3, 7, 14, 21, and 28 days after surgery. There were no significant differences over time (p = 0.44) or between treatment groups (p = 0.51).

The time to reach the platform for each mouse was normalized to its performance on the first day of testing. No significant differences in the time to find the platform were observed between the treatment groups (Fig 4, p = 0.23). Within the No Lens group with cranial windows, one of the mice had markedly longer times to find the platform at both the -14 Day and 3 Day time points (please refer S1 Dataset). In contrast, the mean times of the sham surgery and GRIN lens implant groups were similar. Swim speeds (Fig 5) were nearly unchanged throughout the entire testing period (effect of time, p = 0.44) and there was no difference between treatment groups (p = 0.51).

### *In vivo* imaging

**Fig 6:**
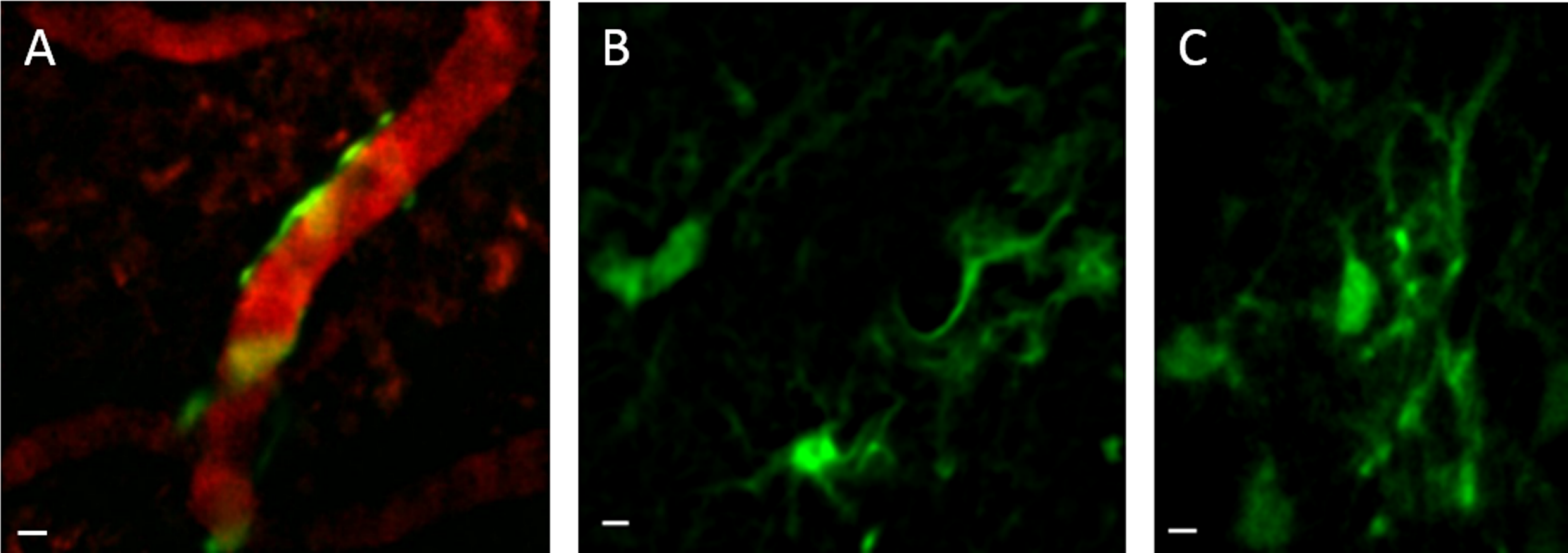
*In vivo* images of glial cells in cortical layer 5. Glial cells in layer 5 of the prefrontal cortex of GFAP-GFP mice imaged using implanted ultrathin GRIN lenses and multiphoton microscopy. Digital zoom images were acquired to show details of cells; the full field of view was 180 μm. **A.** Glial cells expressing GFP under the GFAP promoter are wrapped around a blood vessel counterstained with dextran-Texas red dye. This image is a z-projection of 3 sequential image planes with a 1-μm step size. **B.** This image is a z-projection of 2 image planes, which was done to average out noise and to better visualize cellular processes. **C.** Single plane image of glial cells in a different mouse. Scale bars denote 5 μm.

**Fig 7:**
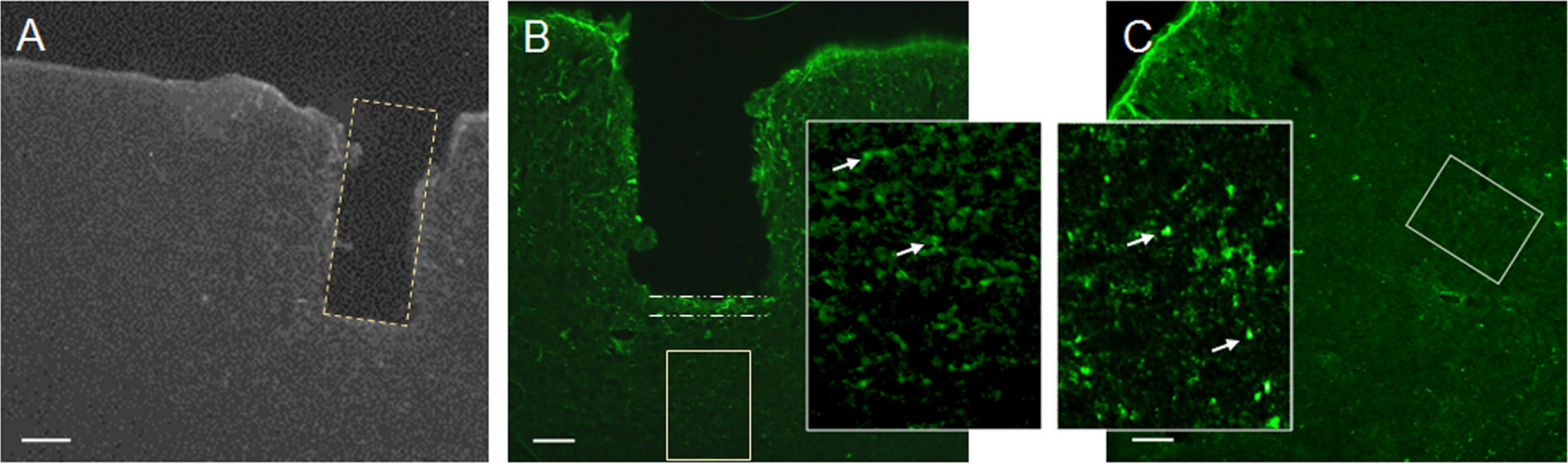
Placement of lens and measurement of glial scar. **A.** Coronal slice containing the track of an implanted GRIN lens is outlined with a dotted yellow line. The implant depth was 935 μm below the pial surface. **B.** Coronal slice stained with anti-GFAP antibody. The white dashed lines denote the mean thickness of the glial scar under the lens, which was 45.9 ± 4.2 μm (mean ± SEM, 40 areas, 3 mice). The working distance of the GRIN lens, which was 125 μm, is much longer than the thickness of the scar. Therefore, the cells within 125 μm of the lens are too close for inclusion in any *in vivo* images. A solid light yellow line denotes the region that can be visualized with the GRIN lens and multiphoton microscopy (imaging region). The GFAP+ cells in the imaging region were relatively few and exhibited only weak immunoreactivity compared with the scar. The inset is an enlargement of this area (inset was brightened and contrast was increased to show relatively faint staining). **C.** An area in the ventrolateral cortex of the contralateral hemisphere was used as a negative control. A solid light yellow line denotes the region which is at the same depth and size as the imaging region and in the same orientation with respect to the pial surface. The inset is an enlargement of this area (inset was brightened and contrast was increased to show relatively faint staining). The number of GFAP+ cells were not significantly different for the imaged region and the negative control region (p = 0.36). Scale bars are 200 μm for A and 100 μm for B and C.

Implantation of 350-μm diameter ultrathin GRIN lenses in mouse prefrontal cortex allowed the capture of images of glial cells below cortical layer 4 in a live mouse for the first time (Fig 6). These images, collected with a multiphoton microscope, show glial cells and their processes in layer 5 of the prefrontal cortex of GFAP-GFP mice [33]. An advantage of implanted GRIN lenses is the ability to perform longitudinal studies involving the *in vivo* monitoring of cells over long periods of time, as demonstrated by representative images acquired 2 months and 14 days after surgery, respectively (Fig 6B and 6C). Two-channel acquisition permits vascular counterstaining for establishing landmarks for longitudinal imaging (Fig 6A). Coronal sections of the prefrontal cortex of implanted mice reveal an approximate depth of 0.9 mm for the implanted lens (Fig 7A).

### Assessment of gliosis after lens implant

Brain slices that included the lens track were immunostained with anti-GFAP antibodies to visualize the glial scar that developed around the lens (Fig 7B). The thickness of the glial scar was 45.9 ± 4.2 μm (mean ± SEM of 40 areas under the lens track in 3 mice; this dimension is outlined by the white dashed lines). The number of GFAP+ astrocytes (mean ± SEM) in the area under the scar region, which included the imaging region, were not significantly different from a control region (Fig 7C) in the ventrolateral cortex of the contralateral hemisphere (4.83 ± 0.49 vs. 5.19 ± 0.58 μm GFAP+ cells per area, respectively: p = 0.36). This control region was chosen because of the relative sparsity of reciprocal connections between it and the area of implant and thus lower likelihood of retrograde neuronal degeneration which could cause distant gliosis. The number of cells in the scar were not counted because the higher density of cells in this area made it difficult to do so unambiguously.

**Fig 8:**
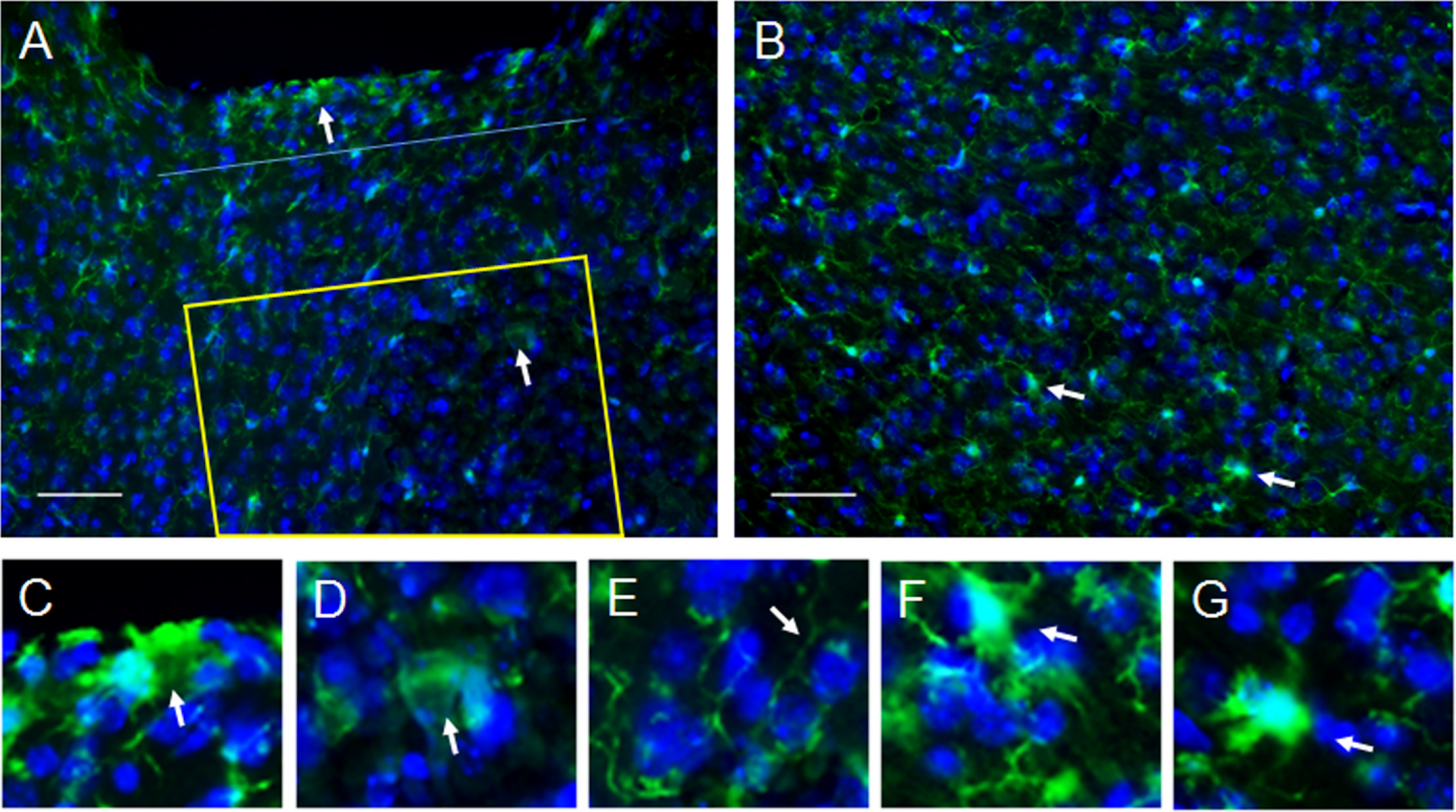
Assessment of microglia morphology. **A.** Several microglial cells had de-ramified or amoeboid morphology, typical of activated microglia, in the non-imaging area up to 125 μm from the end of the lens (cell identified by upper arrow is enlarged in **C**) with 85.7% of these occurring in the same area as the glial scar (n = 3 mice). The dark area along the upper edge of the image is the lens track. A blue dotted line is placed 46 μm from the bottom of the lens track which represents the mean thickness of the glial scar. Only a few activated microglia (cell identified by lower arrow is enlarged in **D**) were observed in the imaging areas, which is illustrated using a yellow outline above. Most microglia had a ramified morphology which is indicative of the resting state (arrow in **E** points to ramified process). **B.** Similar to the region under the lens, the control areas had very few microglia with activated morphology (cells identified by arrows are enlarged in **F** and **G**). Scale bars are 50 μm.

In addition to the upregulation of GFAP in the glial scar region under the lens, the immune response from resident microglial cells was assessed. Microglia were identified by immunostaining for Iba1. As expected, microglia in the resting state with ramified morphology were observed in all 3 areas assessed for GFAP+ cells, including the non-imaged area, the imaging area, and the control area in the ventrolateral cortex in the contralateral hemisphere (Fig 8A, 8B and 8E). However, a few activated microglia with de-ramified and amoeboid morphologies were also observed in all three regions (examples are identified by white arrows in Fig 8A and 8B and enlarged in 8C, 8D, 8F and 8G). A significantly higher number of activated glial cells (2.2 x 10^−4^ ± 4.2 x 10^−5^ cells/μm^2^ mean SEM, 3 implanted mice) was observed up to 125 μm from the bottom of the lens track (non-imaging area) compared to the area beyond it which is the imaging region (2.3 x 10^−5^ ± 1.2 x 10^−5^ cells/μm^2^, p = 0.0106). Notably, 85.7% of the microglia with activated morphology were located within 46 μm from the bottom of the lens, which is the mean thickness of the glial scar (denoted by a blue line in Fig 8A). Similarly, the number of activated microglia in the non-imaged area was greater than the number in the control area (2.5 x 10^−5^ ± 5.1 x 10^−6^ cells/μm^2^, p = 0.0114). In contrast, there was no difference between the number of cells with activated morphology in the imaging area and the control area (p = 0.875).

## Discussion

### Utility of ultrathin GRIN lenses for longitudinal and multimodal imaging studies using mouse models

Our understanding of the brain has deep roots in animal research. Rodents have played a prominent role in this effort. For example, mice and rats have been used for behavioral tests, histology, electrophysiology and other techniques to elucidate mechanisms ranging from brain development to the progression of neurodegenerative diseases. Mice in particular have been useful in combined mode techniques such as (1) multiphoton microscopy of cortical calcium dynamics in behaving animals and (2) optogenetics paired with *in vivo* electrical recordings. Combined mode studies have provided high content data sets and enhanced our knowledge of brain network function in healthy mice and in mouse models of human disease and injury [13, 44].

Micro-optic implants, such as GRIN lenses, are well suited for combined mode, longitudinal imaging studies in mouse brain regions that are too deep to reach through standard multiphoton microscopy. These lenses provide subcellular spatial resolution and temporal discrimination of cellular and microvascular activity as low as the millisecond time range, which noninvasive imaging modalities do not provide [19]. Furthermore, implanted animals could be used for long-term behavioral experiments, electrophysiology, biomarker measurements, and physiological tests. Such combined mode studies would enable an unprecedented level of comparison of multiple factors and observation of potential interactions over time. In essence, each mouse would become a “living laboratory.” This ability to more accurately correlate one effect with another will lead to the identification of cellular and network mechanisms involved brain function and behavior in less time, with lower costs, and with greater confidence.

### Rationale for lens implantation in the frontal cortex

We chose the prefrontal cortex at the interface of the frontal association cortex and motor cortex because this region has neuronal projections to numerous distant networks that affect a wide variety of behaviors. Due to these connections, this region is involved in memory and motor planning functions [45, 46] and performance of motor tasks [47]. The frontal association cortex has been studied in a wide variety of rodent models of human disease, including epilepsy [48], addiction [46, 49, 50], and aging [51]. Because this region is involved in memory, motor planning, and motor tasks we chose commonly used mouse behavioral tasks that assess these functions. These tests were rotarod, foot fault (grid walking), and Morris water maze tests which assessed integrated motor ability, locomotor ability, and spatial learning and memory, respectively [32]. In the present study, we found no differences between the performance of animals with a GRIN lens implant versus those with a cranial window or a sham surgery in any of the four performance measures. This paves the way for combining these behavioral tests with *in vivo* imaging for longitudinal studies using an ultrathin GRIN lens implanted in the prefrontal cortex of mice.

### Rationale for using a thinner, singlet GRIN lens

Compound lenses that are 500-microns in diameter and larger are sheathed to hold the multiple lenses together which increases their size, although the literature generally only reports the lens diameter [14, 52]. They require aspiration of brain tissue prior to implantation. If aspiration is not performed, a relatively large amount of dead cells accumulates beneath the lens during implantation and this deforms the underlying tissue that did not occur with the ultrathin lenses (pers. observation). Notably, aspiration of cortical tissue has been used as a model of brain injury [53, 54]. This is a confounding factor that we do not wish to introduce into our experiments. For these reasons, we decided to use ultrathin, 350-μm diameter, singlet lens. Additionally, by using a singlet lens we have a four- to five-fold greater field of view than the larger diameter compound lenses [18].

### Immune reaction was not evident in imaging region

A glial scar forms around any device chronically implanted in the brain, including microelectrode arrays [55], GRIN lenses [56], dialysis probes and similar probes [57]. As expected, the ultrathin GRIN lenses implanted in the present study elicited a glial scar. This scar had a mean thickness of 46 μm under the lens, which is slightly larger than the thickness of the glial scar that formed under larger micro-optic implants, which was between 25–40 microns thick ([56] in the author’s supplemental data). In that study, the device was implanted on top of the corpus callosum, which may have resulted in the slightly thinner scar. The working distance of the GRIN lenses used in this study was 125 μm, which means that the closest tissue that the lens can image is 125 μm from the implanted end of the lens. In other words, given a glial scar that is 46 μm thick, the closest image to the end of the lens that our system can acquire is 79 μm past the scar layer. As such, the scar tissue does not appear in the images. Furthermore, the scar under the lens does not appear to affect the tissue in the region that is imaged because the number of GFAP+ cells and activated microglia was very low and resembled the numbers of GFAP+ and activated microglia in the undamaged control region in the ventrolateral cortex of the contralateral hemisphere.

### Considerations for generalizing the findings

It should be noted that the lens used in this experiment is 350-μm in diameter which markedly thinner than GRIN lens assemblies and prisms most commonly used for *in vivo* brain imaging, which have diameters of 1 mm and larger [56, 58]. Additionally, microendoscope systems using GRIN lenses are 1.5 mm and more in diameter [30]. Due to this relatively large size, most GRIN lens implants require aspiration of the overlying cortex to reach their target regions [30, 56]. Additionally, implanted prisms compact a rather large area of the cortex [58]. In contrast, the 350-μm diameter lens that we implanted does not require aspiration and displaces less than one quarter the tissue of these other devices. Thus, the absence of an effect on performance in this study may be related to the ultrathin profile of our lens system and lack of tissue aspiration. As such, our results might not be generalizable to all micro-optic implants. The lack of any behavioral effects using our ultrathin lens is not surprising because the diameter of the implant is only slightly larger than one cortical column [59] resulting in a relatively small perturbation of tissue.

### Each animal served as its own control reducing animal numbers

As demonstrated in the present study, an additional benefit of employing a longitudinal approach is the ability to use an animal’s initial behavioral performance as its own control. The animal’s performance before the induction of a condition, such as an injury, a conditional knockout, the administration of a therapeutic drug, or other procedure can be used to normalize that animal’s performance after induction of a desired condition. Normalization minimizes the effect of variability between animals. Furthermore, using the same animals for each time point, as was done in the present study, requires far fewer animals than using separate cohorts for each time point. Reducing experimental variability and utilizing the same animals throughout a study will decrease the number of animals required for experiments.

## Conclusion

In conclusion, implantation of an ultrathin GRIN lens into the prefrontal cortex of mice did not have any measurable effects on vestibulomotor and spatial memory tests. This finding clears the way for performing longitudinal, combined mode studies that include these behavioral tests and *in vivo* imaging of the prefrontal cortex using ultrathin lenses. Furthermore, it is possible that implantation in other cortical areas would also not affect performance on these behavioral tests due to minimal disruption of tissue. It would take relatively few mice and a short amount of time to determine if this is the case for other regions. Using implanted, ultrathin GRIN lenses in longitudinal, combined mode studies will enhance preclinical tests of candidate therapeutics for future human use. Furthermore, this approach will enable more nuanced studies of the structure and function of cells, local networks, and vasculature in mouse models of neurological disorders, development, aging, and injury.

## Supporting Information

S1 ARRIVE Checklist (PDF)

S1 Dataset Images and spreadsheets used for data analysis are available at http://dx.doi.org/10.6084/m9.figshare.1604948. (DOCX)
