# Supplementary material for: Gradient Index Microlens Implanted in Prefrontal Cortex of Mouse Does Not Affect Behavioral Test Performance over Time
Source: PLoS One. 2016 Jan 22;11(1):e0146533. doi: 10.1371/journal.pone.0146533 (PMC4723314; doi:10.1371/journal.pone.0146533)
Supplement: S1 Dataset — (DOCX) [file pone.0146533.s002.docx]

**S1 Dataset**

**Description**

Supporting data list of files with doi <http://dx.doi.org/10.6084/m9.figshare.1604948>

Behavioral data and histograms for Figs 2 – 5 <http://dx.doi.org/10.6084/m9.figshare.1579586>

In vivo image of glial cells for Fig 6A (green) <http://dx.doi.org/10.6084/m9.figshare.1604942>

In vivo image of glial cells for Fig 6A (red) <http://dx.doi.org/10.6084/m9.figshare.1604942>

In vivo image of glial cells for Fig 6B <http://dx.doi.org/10.6084/m9.figshare.1579584>

In vivo image of glial cells for Fig 6C <http://dx.doi.org/10.6084/m9.figshare.1579583>

Lens track in coronal slice for Fig 7A <http://dx.doi.org/10.6084/m9.figshare.1579590>

Lens track in coronal slice, outlined for Fig 7A <http://dx.doi.org/10.6084/m9.figshare.1579591>

GFAP stained brain slice B5M2 with lens track for Fig 7B <http://dx.doi.org/10.6084/m9.figshare.1579589>

GFAP stained brain slice B5M2 control region for Fig 7C <http://dx.doi.org/10.6084/m9.figshare.1579588>

GFAP stained brain slice B3F2 lens track region <http://dx.doi.org/10.6084/m9.figshare.2036934>

GFAP stained brain slice B3F2 control region <http://dx.doi.org/10.6084/m9.figshare.2037540>

GFAP stained brain slice B5M4 lens track region <http://dx.doi.org/10.6084/m9.figshare.2037744>

GFAP stained brain slice B5M4 control region <http://dx.doi.org/10.6084/m9.figshare.2037885>

Activated microglia counts <http://dx.doi.org/10.6084/m9.figshare.2039616>

M3E2 activated microglia under lens <http://dx.doi.org/10.6084/m9.figshare.2041911>

M3E2 activated microglia control region <http://dx.doi.org/10.6084/m9.figshare.2042439>

M1F4 activated microglia under lens <http://dx.doi.org/10.6084/m9.figshare.2042634>

M1F4 activated microglia control region <http://dx.doi.org/10.6084/m9.figshare.2042802>

M3F3 activated microglia under lens <http://dx.doi.org/10.6084/m9.figshare.2042967>

M3F3 activated microglia control region <http://dx.doi.org/10.6084/m9.figshare.2043105>
